# LPS Tolerance Inhibits Cellular Respiration and Induces Global Changes in the Macrophage Secretome

**DOI:** 10.3390/biom11020164

**Published:** 2021-01-27

**Authors:** Joseph Gillen, Thunnicha Ondee, Devikala Gurusamy, Jiraphorn Issara-Amphorn, Nathan P. Manes, Sung Hwan Yoon, Asada Leelahavanichkul, Aleksandra Nita-Lazar

**Affiliations:** 1 Functional Cellular Networks Section, Laboratory of Immune System Biology, National Institute of Allergy and Infectious Diseases, National Institutes of Health, Bethesda, MD 20892-1892, USA; joseph.gillen@nih.gov (J.G.); nathan.manes@nih.gov (N.P.M.); sunghwan.yoon@nih.gov (S.H.Y.); 2 Department of Microbiology, Faculty of Medicine, Chulalongkorn University, Bangkok 10330, Thailand; thunnichaon@yahoo.com (T.O.); jiraphorn298@gmail.com (J.I.-A.); aleelahavanit@gmail.com (A.L.); 3 Surgery Branch, National Cancer Institute, Bethesda, MD 20892-1892, USA; devikala.gurusamy@nih.gov; 4 Translational Research in Inflammation and Immunology Research Unit (TRIRU), Department of Microbiology, Chulalongkorn University, Bangkok 10330, Thailand

**Keywords:** host-pathogen interactions, proteomics, secretome, macrophages

## Abstract

Inflammatory response plays an essential role in the resolution of infections. However, inflammation can be detrimental to an organism and cause irreparable damage. For example, during sepsis, a cytokine storm can lead to multiple organ failures and often results in death. One of the strongest triggers of the inflammatory response is bacterial lipopolysaccharides (LPS), acting mostly through Toll-like receptor 4 (TLR4). Paradoxically, while exposure to LPS triggers a robust inflammatory response, repeated or prolonged exposure to LPS can induce a state of endotoxin tolerance, a phenomenon where macrophages and monocytes do not respond to new endotoxin challenges, and it is often associated with secondary infections and negative outcomes. The cellular mechanisms regulating this phenomenon remain elusive. We used metabolic measurements to confirm differences in the cellular metabolism of naïve macrophages and that of macrophages responding to LPS stimulation or those in the LPS-tolerant state. In parallel, we performed an unbiased secretome survey using quantitative mass spectrometry during the induction of LPS tolerance, creating the first comprehensive secretome profile of endotoxin-tolerant cells. The secretome changes confirmed that LPS-tolerant macrophages have significantly decreased cellular metabolism and that the proteins secreted by LPS-tolerant macrophages have a strong association with cell survival, protein metabolism, and the metabolism of reactive oxygen species.

## 1. Introduction

Macrophages and monocytes are innate immune cells playing an important role in orchestrating the initial response to bacterial infection and tissue damage [1]. During Toll-like receptor (TLR) stimulation, macrophages are activated and produce pro-inflammatory cytokines and chemokines to recruit other cells to the site of infection [1,2]. In sepsis, lipopolysaccharides (LPS), an outer membrane component of Gram-negative bacteria, are considered to be a major activator of macrophages, triggering an inflammatory response [3]. However, in response to a second or prolonged LPS stimulation, macrophages are initially activated but produce lower amounts of pro-inflammatory cytokines. This phenomenon is called “LPS tolerance” or “endotoxin tolerance” and has been known since the 1940s [4,5,6]. While the lower cytokine production during LPS tolerance prevents a severe “cytokine storm” response and lethal effects in the host, decreased cytokine levels might not be sufficient to maintain an effective defense against pathogens. Indeed, LPS tolerance has been reported to be associated with the immune suppression stage known as immune exhaustion [6]. A concept of innate immunity bearing a memory of past insults termed “trained immunity” encompasses endotoxin tolerance, and its exploration may result in discoveries of new immunotherapies [7].

The mechanisms inhibiting the LPS response and moving cells into a tolerant state have still not been completely elucidated [8]. Findings from several groups emphasize the roles in this process of epigenetic reprogramming [4], microRNA [9,10], alteration of gene expression patterns [11], sometimes by specific transcription factors such as hypoxia-inducible factor 1-alpha (HIF-1α) [12], non-coding RNAs [13], and energy depletion [14]. The metabolic changes in LPS-challenged macrophages after treatment with LPS have been indicated by several recent studies, with varying experimental designs focusing on a specific protein [15], pathway [16], or general phenotype [17]. Regulation of cellular signaling leads to changes in multiple secreted proteins that are responsible for the immune response during TLR stimulation (e.g., interleukin (IL)-6 and tumor necrosis factor (TNF)-α). These proteins act as autocrine, paracrine, or chemoattracting signaling molecules for communication with other immune cells [18]. We have recently demonstrated the role of secreted lipocalin 2 (Lcn2) in the reduction in macrophage cytokine release in LPS-tolerant cells [15], but a comprehensive secretome analysis of LPS tolerance has not been previously reported. Investigating the secretome during tolerance induction could provide directions for explaining the phenomenon of immune tolerance and exhaustion.

In this study, we used metabolic measurements to confirm differences in the cellular metabolism of naïve macrophages and that of either macrophages responding to LPS stimulation or macrophages in the LPS-tolerant state. Next, we used mass spectrometry-based proteomics to thoroughly investigate, for the first time, the changes in the extracellular proteome (secretome) following the induction of LPS tolerance. Furthermore, we investigated the secretome profile during the induction of LPS tolerance to identify possible regulators of cellular metabolism and the production of proteins. In our analysis, we confirmed that LPS-tolerant macrophages have significantly decreased cellular metabolism and that the proteins secreted by LPS-tolerant macrophages have a strong association with cell survival, protein metabolism, and reactive oxygen species metabolism.

## 2. Materials and Methods

### 2.1. Cell Culture and Reagents

RAW 264.7 mouse macrophage cells were cultured in Dulbecco’s Modified Eagle’s Medium (DMEM) supplemented with 10% fetal bovine serum(FBS), 1 × glutamine, and 20 mM 4-(2-hydroxyethyl)-1-piperazineethanesulfonic acid (HEPES) buffer, referred to as complete DMEM (cDMEM). For stable isotope labeling by amino acids in cell culture (SILAC), the cells were cultured in DMEM for SILAC purchased from Thermo Fisher Scientific (Waltham, MA, USA), supplemented with 10% FBS, 1 × glutamine, 20 mM HEPES buffer, and isotopically labeled lysine and arginine purchased from Cambridge Isotope Laboratories, Inc. (Tewksbury, MA, USA). The cells were cultured in the labeled media for five passages prior to analysis to allow for > 95% incorporation of the labeled amino acids. Lipopolysaccharide (LPS) from *Salmonella minnesota* R595 was purchased from Enzo Life Sciences, Inc. (Farmingdale, NY, USA).

### 2.2. Quantification of Secreted Cytokines

Secreted TNF-α, IL-6, and IL-10 were quantified with ELISA kits (Thermo-Scientific, Rockford, IL, USA) following the manufacturer’s protocols.

### 2.3. Extracellular Flux Analysis

The energy metabolism profiles of macrophages can be used to estimate glycolysis and mitochondrial oxidative phosphorylation on the basis of the extracellular acidification rate (ECAR) and the oxygen consumption rate (OCR), which were measured using Seahorse XF Analyzers (Agilent, Santa Clara, CA, USA). RAW 264.7 cells in different experimental groups, namely untreated (NT/NT or Con), LPS-responsive (NT/LPS or LR), and LPS-tolerant (LPS/LPS or LT), were dispersed into monolayers for measurement. A RAW mitochondrial stress test and a glucose stress test were performed at 37 °C using the Seahorse XFe96 bioanalyzer (Agilent, Santa Clara, CA, USA). RAW 264.7 cells in various treatment groups were collected and washed in 1× PBS. Cells seeded at 4 × 10^5^ cells per well of the Seahorse analysis plates were centrifuged at 400 rpm with acceleration and deceleration set to 1 for 5 min to achieve an even monolayer of cells for accurate measurement. OCR and ECAR for the mitochondrial stress test were measured in xeno-free (XF) media (containing 25 mM glucose, 2 mM L-glutamine, and 1 mM sodium pyruvate) under basal conditions and in response to 2 μM oligomycin, 1.5 μM fluoro-carbonyl cyanide phenylhydrazone (FCCP), and 0.5 μM rotenone and antimycin A (Sigma-Aldrich, St. Louis, MO, USA). For the glucose stress test, the cells were cultured in XF media (containing 2 mM L-glutamine), and the ECAR readout was obtained at basal conditions and in response to 10 mM glucose, 1 µM oligomycin, and 10 mM 2-deoxy-glucose (2-DG).

### 2.4. Collection of Secreted Proteins

For the secretome analysis by quantitative liquid chromatography–tandem mass spectrometry (LC-MS/MS), we used the method we established earlier [19]. Briefly, prior to stimulation, 1 × 10^6^ RAW 264.7 cells were seeded in a well of a 12-well plate and grown at 37 °C for 24 h. To decrease the amount of non-specific protein in the media prior to stimulation, the cDMEM was removed from the cell culture wells and replaced with cDMEM lacking FBS. We extensively evaluated cell death in this method and found it to be negligible [19]. To test the effect of multiple stimulations of the innate immune system, the RAW 264.7 cells were treated in one of three ways. The first group contained control cells that received no LPS (NT/NT or Con) and were grown in cDMEM labeled with Arg^0^ and Lys^0^. The second group received a single stimulation with 100 ng/mL LPS 6 h prior to the collection of the media (NT/LPS or LR) and were grown in cDMEM labeled with Arg^+6^ and Lys^+4^. The third group received two stimulations with 100 ng/mL LPS separated by 24 h, with the second stimulation being 6 h prior to the sample collection (LPS/LPS or LT), and were grown in cDMEM labeled with Arg^+10^ and Lys^+8^. After the stimulations, the media were collected and equal parts of the three groups (*v*/*v*, as in [19,20,21]) were combined into a single 1.5-mL tube. Any cellular debris or detached cells were separated from the media by filtration using a 0.22-µm polysaccharide filter, and then, the medium was centrifuged at 400× *g* for 5 min. Finally, the supernatant was transferred to a 1.5-mL tube and the proteins were concentrated in a vacuum centrifuge (SpeedVac, Thermo Fisher Scientific, Waltham, MA) to dryness. Overall, this method was repeated twice with two biological replicates each time to produce four biological replicates.

### 2.5. In-Gel Digestion of Secreted Proteins

The dried proteins were resuspended in 2 × NuPAGE loading buffer, and then, the proteins were denatured by boiling for 10 min. The proteins were separated using a 10% Bis-Tris NuPAGE gel (Invitrogen, 8 × 8 cm) with 3-(N-morpholino)propanesulfonic acid (MOPS) buffer and run with 200 V for 40 min to ensure that there were no significant visual differences in the band patterns between samples. The gel was fixed using 47.5% methanol and 5% glacial acetic acid for 30 min at room temperature and then washed three times with ddH_2_O. The fixed proteins were stained with PageBlue protein staining solution (Thermo Fisher Scientific, Waltham, MA, USA) for 1 h at room temperature and then destained with ddH_2_O overnight at 4 °C. Following destaining, the lanes were cut from the gel using razor blades, sectioned into five equal units to avoid processing excess gel in one sample, and cubed into approximately 1-mm^3^ pieces. The gel pieces from each section were collected into 1.5-mL microcentrifuge tubes and then processed according to a previously published protocol [22].

In brief, 500 µL of acetonitrile (ACN) was added to the gel pieces and the tubes were incubated at room temperature for 10 min before a brief centrifugation and removal of the supernatant. Next, 50 µL of 10 mM dithriothreitol (DTT, Sigma-Aldrich, St. Louis, MO, USA) in 100 mM ammonium bicarbonate (ABC) was added to the gel pieces and the tubes were incubated at 56 °C for 30 min followed by a second incubation with ACN. Next, 50 µL of 55 mM 2-chloroacetamide (CA, Sigma-Aldrich, St. Louis, MO, USA) in 100 mM of ABC was added to the gel pieces and the tubes were incubated at room temperature in the dark for 20 min followed by a third incubation with ACN. Then, 100 µL of 50% ACN, 50 mM ABC was added to the gel pieces and the tubes were incubated at room temperature with occasional vortexing, followed by a fourth incubation with ACN. The gel pieces were saturated with 13 ng/µL sequence-grade modified trypsin (Promega; Madison, WI, USA) in 10 mM ABC, 10% ACN and the tubes were incubated at 37 °C overnight. To extract the peptides, 100 µL of a 1%:25% mix of formic acid:acetonitrile was added to the gel pieces and the tubes were incubated for 15 min in a 37 °C shaker. The tubes were centrifuged briefly and the supernatant was collected in 1.5-mL tubes. At this point, the peptides from the gel sections were recombined to make one sample per lane and the peptides were concentrated in a vacuum centrifuge (SpeedVac, Thermo Fisher Scientific, Waltham, MA, USA). Lastly, the samples were mixed with formic acid and ACN to generate peptide samples with a final concentration of 0.1% formic acid, 2% ACN.

### 2.6. Mass Spectrometry

The Thermo Orbitrap Q-Exactive HF (Thermo Fisher Scientific, Bremen, Germany) and the Thermo UltiMate 3000 systems (Thermo Fisher Scientific, Bremen, Germany) were used for LC-MS/MS experiments. Peptides were trapped on an Acclaim C18 PepMap 100 trap column (5 µm, 100 Å, 300 µm i.d. × 5 mm, Thermo Fisher Scientific, Pittsburgh, PA, USA) and separated on a PepMap RSLC C18 column (2 µm, 100 Å, 75 µm i.d. × 50 cm, Thermo Fisher Scientific, Pittsburgh, PA, USA) at 40 °C. Peptides were eluted with a linear gradient of 2.5% to 5% mobile phase B (0.1% formic acid in ACN) for 15 min and then 5% to 35% mobile phase B over 90 min. Gradient changes were followed at 105 min to 35% mobile phase B and then increased to 99% mobile phase B at 110 min. The gradient was changed back to 2.5% mobile phase B at 125 min to equilibrate for 20 minutes prior to the next injection. Eluted peptides were ionized in positive ion polarity at a 2.3-kV spraying voltage. MS1 full scans were recorded in the range of *m*/*z* 400 to 1600 with a resolution of 60,000 at 200 *m*/*z* using the Orbitrap mass analyzer. Automatic gain control was set at 1 × 10^6^ with 40 ms of maximum injection time. The top 20 data-dependent acquisition mode was used to maximize the number of MS2 spectra from each cycle. Higher-energy collision-induced dissociation (HCD) was used to fragment selected precursor ions with a normalized collision energy of 27%. Each biological replicate was analyzed twice to create two technical replicates. The mass spectrometry-based proteomics data have been deposited to the ProteomeXchange Consortium via the PRIDE partner repository with the dataset identifier PXD021925 [23].

### 2.7. Analysis of MS Results

The RAW MS files were processed with MaxQuant software (version 1.6.5.0, Max Planck Institute, Munich, Germany) [24] and searched with the Andromeda search engine [25] against a mouse UniProt FASTA database (download date: 26.03.2019, 22,325 entries) supplemented with common contaminants and reverse sequences of all entries [26]. The Andromeda search engine parameters were: type = three labels—light (Arg0, Lys0), medium—(Arg6, Lys4), and heavy—(Arg10, Lys8); fixed modification = carbamidomethylation of cysteine; variable modifications = oxidation of methionine, acetylation of lysine, and acetylation of protein N-terminus; minimum peptide length = 7; and max missed cleavages = 2. The false-discovery rate was set to 0.01 at the peptide spectrum matches (PSM), peptide, and protein levels.

The protein group abundance data were filtered to remove possible protein contaminants. In addition, at least 2 identified peptides were required for each protein (if only one peptide was identified, at least 12 valid abundance values were required). This resulted in an estimated protein group false discovery rate of 1.54%. If a SILAC triplet contained one or two missing values, they were imputed by randomly generating a value between 10% and 100% of the minimum protein intensity value (equally distributed; separately for each LC-MS dataset). The abundance ratios were log2-transformed, and mean values were calculated across the technical replicates. InfernoRDN software v1.1.7626.35996 (https://omics.pnl.gov/software/infernordn) [27] was used to perform *t*-tests and to calculate post-hoc *q*-values.

## 3. Results

### 3.1. LPS-Tolerant Macrophages Decrease Cellular Respiration

**Figure 1:**
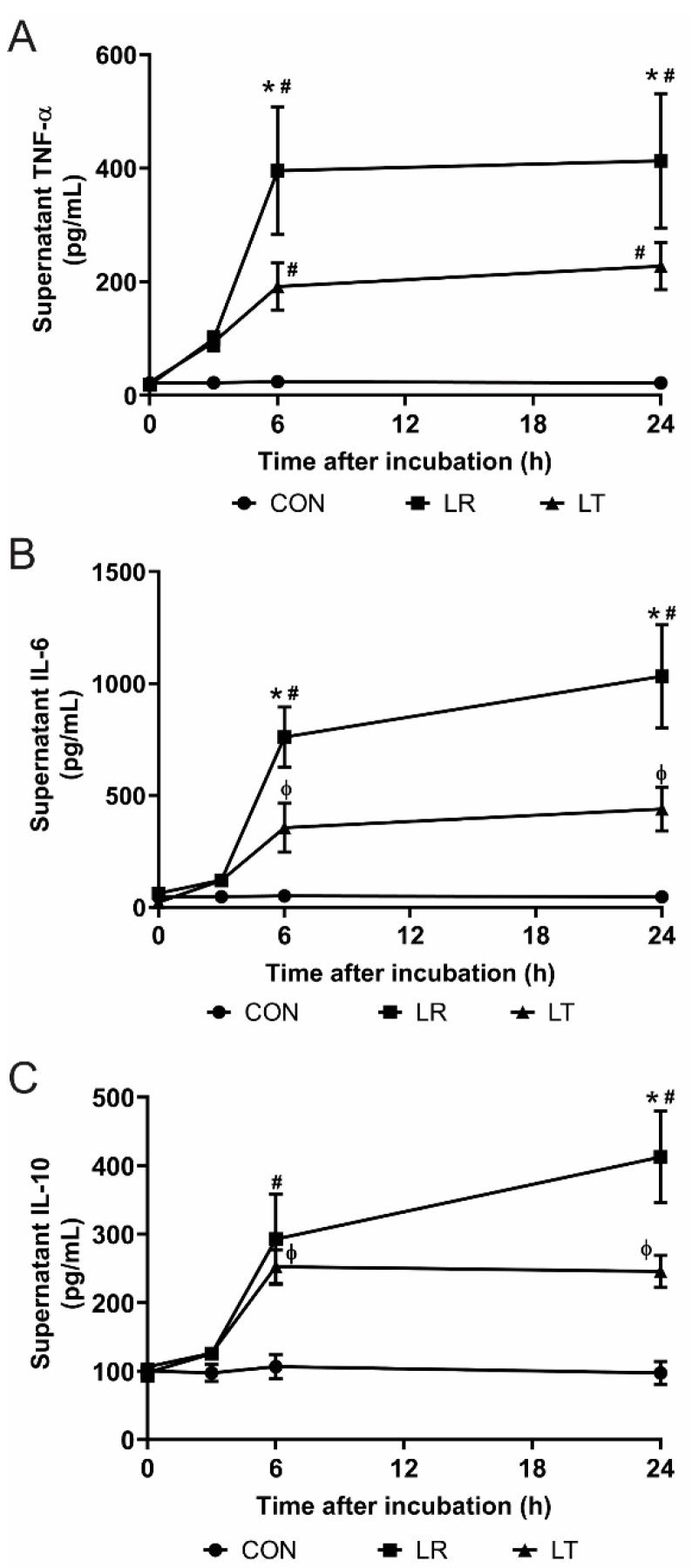
Induction of lipopolysaccharide (LPS) tolerance inhibits cytokine production by RAW 264.7 cells. Inflammatory cytokines in the supernatants (**A**–**C**) (n = 4/time point) of macrophages treated either once (LPS-Responding (LR)) or twice with LPS stimulation (LPS-Tolerant (LT)) and untreated control samples (Con), measured using ELISA kits, show significantly inhibited secretion of tumor necrosis factor (TNF)-α (**A**), interleukin (IL)-6 (**B**), and IL-10 (**C**) from LT cells compared to LR cells. *, *p*-value < 0.05 vs. LR; #, *p*-value < 0.001 vs. Con; ϕ, *p*-value < 0.05 vs. Con.

LPS tolerance is the decreased response of immune cells following secondary or prolonged stimulation with LPS. This process is typified by the decreased secretion of cytokines (Figure 1A–C), but the causes of tolerance and the processes that maintain it are not completely elucidated. This decreased response, although useful as a mechanism preventing a lethal outcome, can have tragic consequences for patients as the decreased secretion of cytokines might often lead to an increase in secondary infections. To examine the causes and regulation of LPS tolerance in macrophages, we used purified LPS to stimulate RAW 264.7 cells as an in vitro model, a methodology successfully used previously for secretome analysis with conclusions on changes in innate immune pathways and cellular metabolism by us [19] and others (for example, [21,28]). We found that while a single LPS stimulation enhanced cytokine release (LPS-Responding (LR)), two sequential LPS stimulations over a 24-h period induced decreased cytokine levels following a 6-h incubation (LPS-Tolerant (LT)) (Figure 1A–C). These results established the conditions required to induce LPS tolerance in RAW cells.

**Figure 2:**
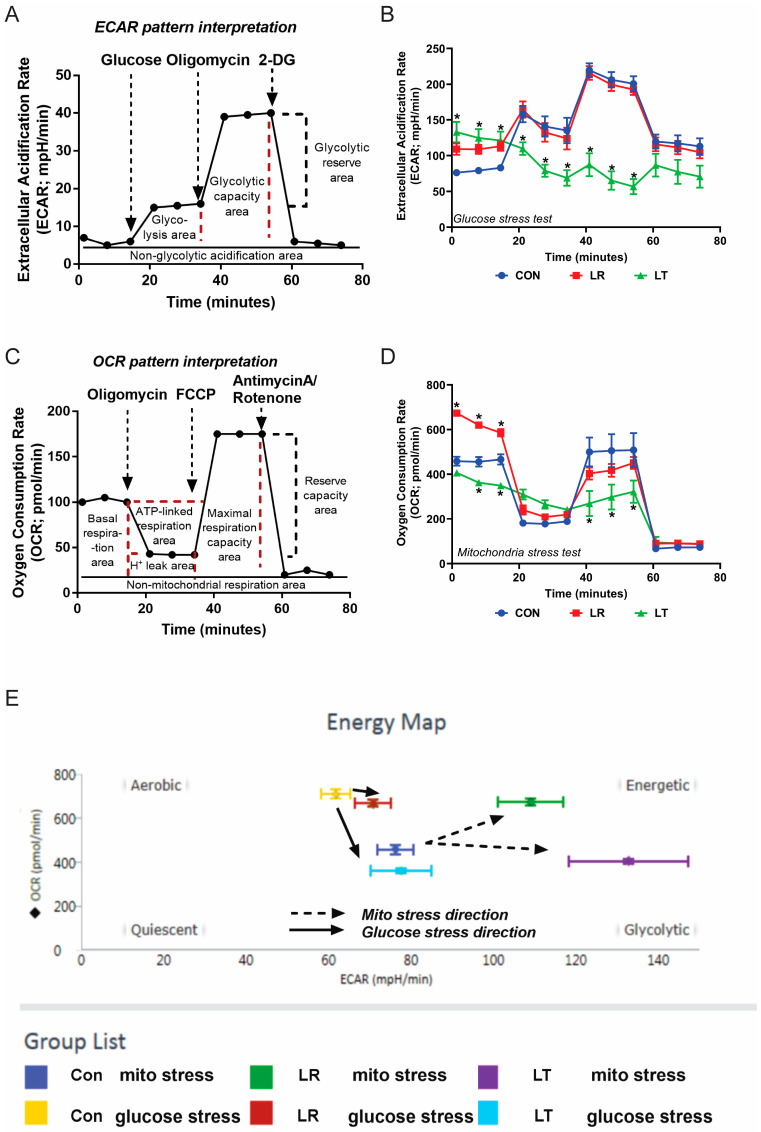
LPS-tolerant RAW 264.7 cells have significantly decreased cellular metabolism compared to LPS-responding and unstimulated cells. The general pattern of the estimation of glycolysis and mitochondrial functions through extracellular acidification rate (ECAR) and oxygen consumption rate (OCR), respectively (**A**,**C**); the pattern of macrophages treated with LPS either once (LPS-Responding (LR, red)) or twice (LPS-Tolerant (LT, green)) and untreated control samples (Con, blue) (**B**,**D**) (combination from triplicate experiments for **B**,**D**), and the energy map calculated using the Seahorse XF Extracellular Flux Assay (**E**). * = *p*-value < 0.001.

While the decrease in secretion could be due to many factors such as a lack of available amino acids to build proteins, inhibition of vesicle transport, or increased turnover of specific mRNAs, we hypothesized that LPS-tolerant cells would display changes in their metabolic functions. The glycolytic and mitochondrial functions of control (Con or NT), LR, and LT cells were determined by measuring the ECAR and OCR using the Seahorse XF Extracellular Flux Analyzer. Both functions were impaired in LT cells compared to Con or LR cells (Figure 2A–E). Hence, the lower macrophage cytokine production in LPS-tolerant cells compared with control cells might be associated with the low cell energy.

### 3.2. Variations in LPS Treatment Lead to Variations in the Secretome

**Figure 3:**
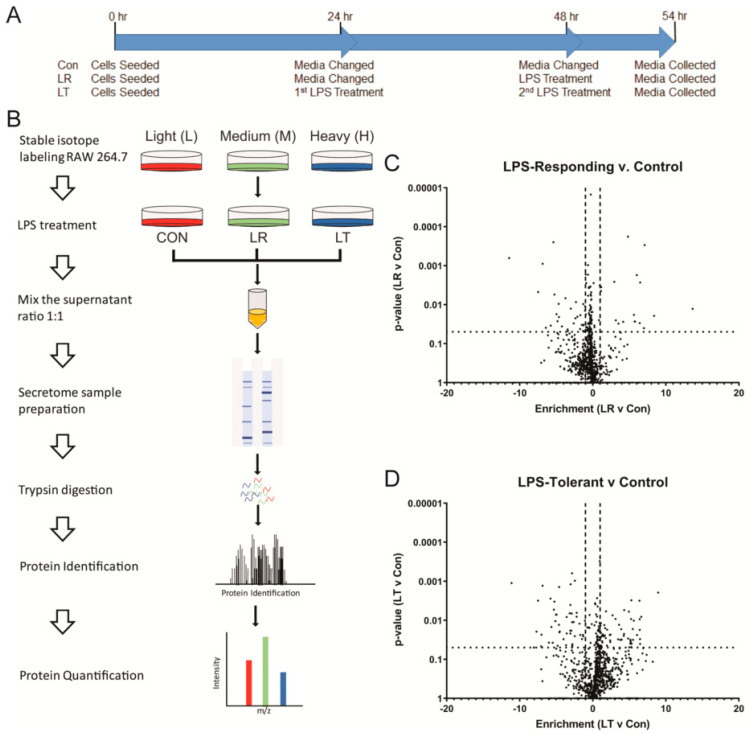
LPS-Responding and LPS-Tolerant RAW 264.7 cells secrete a wide variety of proteins. Schematic of the timing for LPS treatments of RAW 264.7 cells to induce the LPS response (LR) or LPS tolerance (LT) (**A**). MS analysis of the secretome of RAW 264.7 (**B**). Plot of *t*-test results comparing individual protein intensities calculated by MaxQuant (version 1.6.5.0) in LPS-Responding (LR) (**C**) or LPS-Tolerant (LT) (**D**) versus untreated control samples (NT/NT). Protein intensities of eight replicates were averaged and missing values for intrasample results were replaced with a random value between 1/2× and 2× the average of the 10 lowest values. The dotted lines indicate significance (*p*-value < 0.05) and the dashed lines indicate a onefold difference from the control.

To examine the conditions that contributed to the decreased respiration of LT cells, we analyzed the media collected from cells in each condition. By using SILAC metabolic labeling to mark each of the conditions prior to mass spectrometric analysis, as we have done in an earlier analysis of the TLR ligand-induced secretomes [19], we could simultaneously process and quantify the relative amounts of the proteins secreted by the cells in each condition (Figure 3A). We have reliably identified and quantified 1189 proteins across all conditions. Using a *t*-test to compare the intensities of the protein signals identified in the LR or LT samples to the Con samples, we found that several proteins had a two-fold or higher change in relative quantity and a significant change (*p*-value ≤ 0.05) versus the control (Figure 3B,C). In total, we found 56 and 107 proteins with significantly different levels in LR and LT cellular media, respectively.

**Figure 4:**
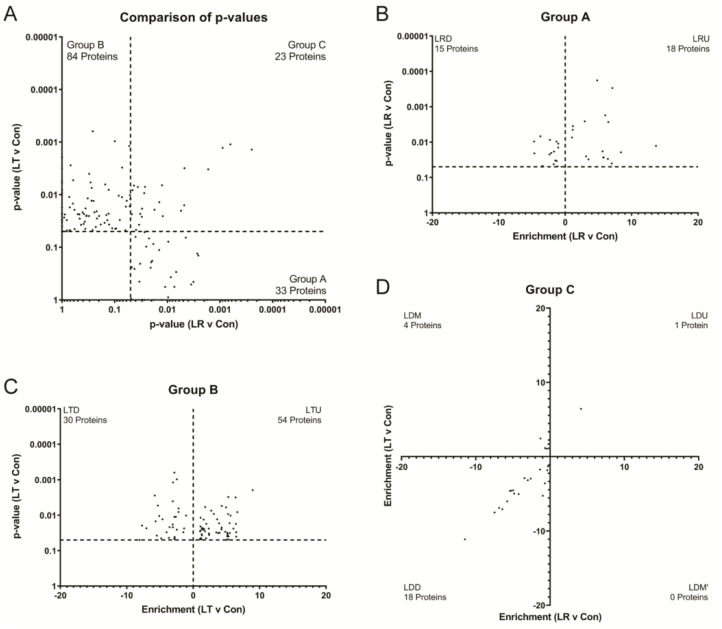
LPS-Tolerant and LPS-Responding RAW 264.7 cells have distinct secretomes. Plotting of *p*-values of LR vs. Con against *p*-values of LT vs. Con confirms that only 19.3% of proteins have significantly modified secretion in both conditions (**A**). Of the proteins with significantly modified secretion in only LR cells, over half of the proteins have increased secretion (**B**). Of the proteins with significantly modified secretion in only LT cells, over half of the proteins have increased secretion (**C**). A comparison of the enrichment of proteins secreted by the LR and LT cells with significantly modified secretion shows that the majority of these proteins were significantly decreased following treatment (**D**).

We found that most proteins were found in both treatment groups but had different levels and directions of change compared to the control in both LR and LT conditions. To identify which changes in protein levels were specific to either LR or LT conditions, we plotted the *p*-values versus the control of each protein in the LR and LT datasets (Figure 4A). This plot identified four clear groups, identified as A through D. Group A proteins had a significant difference (*p*-value ≤ 0.05) in LR samples and included 33 proteins. Group B had a significant difference (*p*-value ≤ 0.05) in LT samples and included 84 proteins. Group C had a significant difference (*p*-value ≤ 0.05) in samples treated with either LR or LT samples and included 23 proteins. Group D had no significant difference following LPS treatment and included 608 proteins (Figure 4A).

In addition to the significant difference from the control, the individual protein results could be further sorted by whether the intensity increased or decreased in comparison to the control (Figure 4B,C; Supplementary Table S1). The proteins that presented increased intensity following LPS treatments were termed subgroup one, while those with decreased intensity were termed subgroup two. Of the group A proteins, 18 were significantly increased (group “LPS-Responding Up” (LRU)) and 15 were significantly decreased (group “LPS-Responding Down” (LRD)). Group B proteins, while more numerous than those in group A, still had a bias towards increasing intensities, with 54 proteins from the group “LPS-Tolerant UP” (LTU) against 30 proteins from the group “LPS-Tolerant Down” (LTD). Lastly, for group C, significance following LPS treatment could lead to three possible outcomes: increased in both conditions (one protein), decreased in both conditions (18 proteins), or a discordant result with increased in one condition but decreased in the other condition (four proteins).

Amongst the proteins in the LRU group were several cytokines and chemokines (Supplementary Table S1), including C-C motif chemokine 4 (Ccl4), tumor necrosis factor (TNF), C-X-C motif chemokine 10 (Cxcl10), C-C motif chemokine 2 (Ccl2), and leukemia inhibitory factor (LIF), which all showed significant (*p*-value < 0.05) or highly significant (*p*-value < 0.001) increases in their average intensity when compared to either the Con or the LT treatment group (Figure 4B, Supplementary Table S1). These data provide a perfect quality control for our dataset because these cytokines and chemokines are essential for the inflammatory response and are expected to be elevated in response to LPS.

The group LRD included three proteins (Beta-glucuronidase (Gusb), beta-hexosaminidase, subunit alpha (Hexb), and alpha-N-acetylglucosaminidase (Naglu)) that localize to the phagolysosome and are associated with the metabolism of carbohydrates [29]. These proteins, along with vinculin (Vin), Dipeptidyl peptidase 2 (Dpp7), and malate dehydrogenase mitochondrial (Mdh2), displayed between a 1.25- and 5.75-fold significant decrease in intensity with *t*-test *p*-values ranging from 0.007 to 0.0484 following the LPS treatment (Supplementary Table S1).

In contrast to the LR groups, the LT groups both contained many proteins typically found in the cytoplasm or other regions of the cell in addition to some secreted proteins (Supplementary Table S1). LTU proteins included 54 proteins with increases ranging from 48- to 2.78-fold versus the control sample and included osteopontin (Spp1), neutrophil gelatinase-associated lipocalin (Lcn2), sequestosome-1 (Sqstm1), and TAR DNA-binding protein 43 (Tarbp) (Figure 4C). The *t*-test *p*-values of each protein versus the control ranged from 0.0491 to 0.0001 (Supplementary Table S1). In contrast to the LTU proteins, nearly half (7/19) of the LTD proteins were associated with extracellular space. The 30 proteins from the LTD group showed between a 22- and 1.6-fold significant (*p*-values between 0.04 and 0.00001) decrease in overall intensity versus the control cells and included the urokinase-type plasminogen activator (Plau), sodium/potassium-transporting ATPase subunit gamma (Fxyd2), lysozyme C-2 (Lyz2), and cystatin-C (Cst3) (Supplementary Table S1).

The last and smallest group of proteins that showed significant differences versus the control depending on the treatment with LPS were group C proteins (LPS-Dependent (LD)). The inclusion of the second treatment group leads to three possible results: both increase (LDU), both decrease (LDD), or one increases and one decreases (mixed) (LDM). In our analysis, we found only one LDU protein, plasminogen activator inhibitor 1 (Serpine1), and four LDM proteins, Talin-1 (Tln), MARCKS-related protein (Marcksl1), cytosolic non-specific dipeptidase (Cndp2), and eukaryotic initiation factor 4A-I (Eif4a1), with increases in at least one treatment group by 29- to 1.6-fold versus the control set (Supplementary Table S1). The last group of 18 proteins identified in our analysis were the proteins with significant decreases in intensity (between 2000- and 1.5-fold) versus the control in both treatment conditions (group LDD), such as gelsolin (Gsn), low-density lipoprotein receptor-related protein 1 (Lrp1), macrophage colony-stimulating factor 1 receptor (Csf1r), and fibronectin (Fn1) (Supplementary Table S1).

### 3.3. Pathway Analysis of Critical Groups

**Figure 5:**
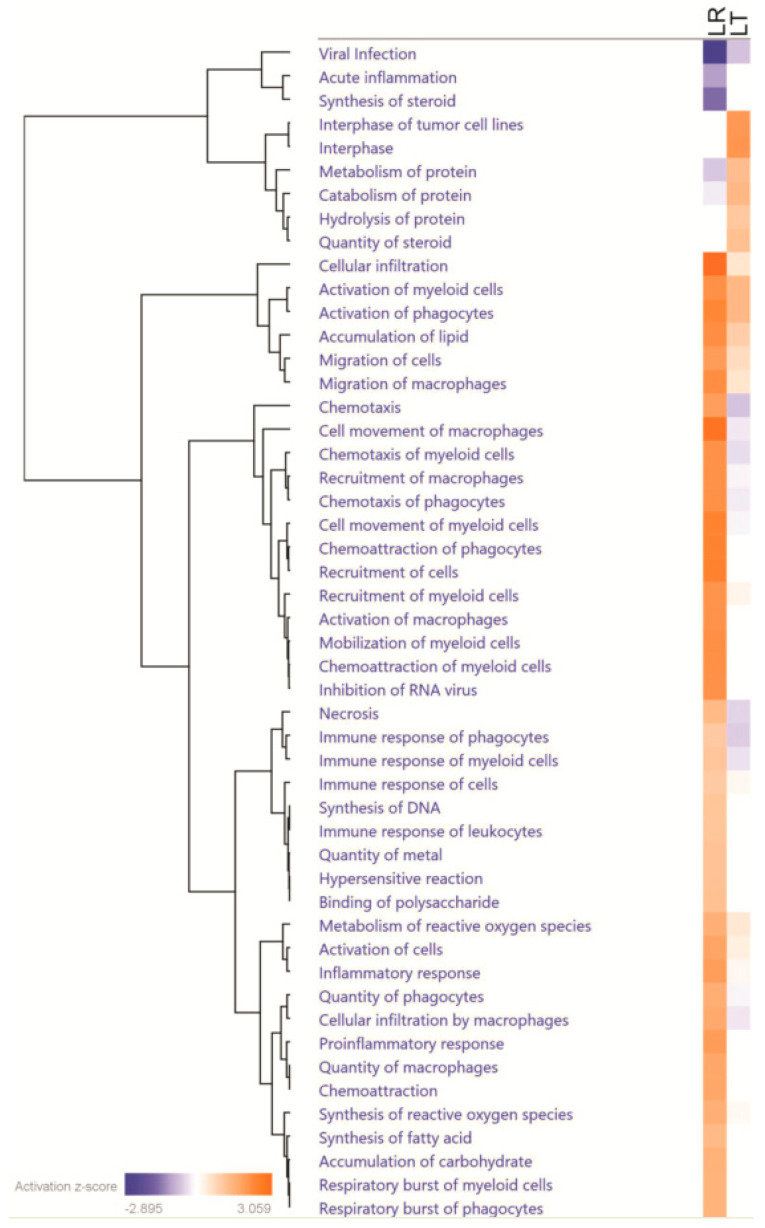
LPS-Responding RAW 264.7 cells have secretomes strongly associated with the immune response in contrast to LPS-Tolerant RAW 264.7 cells. Comparison of the Ingenuity Pathway Analysis of the proteins with significantly changed secretion in either LR or LT cells shows that while LR cells secreted proteins that strongly relate to the immune response and chemotaxis, LT cells secreted proteins that strongly relate to metabolism and cellular survival. Prepared using the Ingenuity Pathway Analysis program suite from QIAGEN (Germantown, MD, USA).

Because either increasing or decreasing secretion of a signaling protein could have profound effects on the condition of cells, we analyzed all proteins with significant changes using the Ingenuity Pathway Analysis (IPA) software suite (Qiagen) (Figure 5). This analysis allowed us to identify several patterns, including pathways or functions enriched in either both or only one dataset.

**Figure 6:**
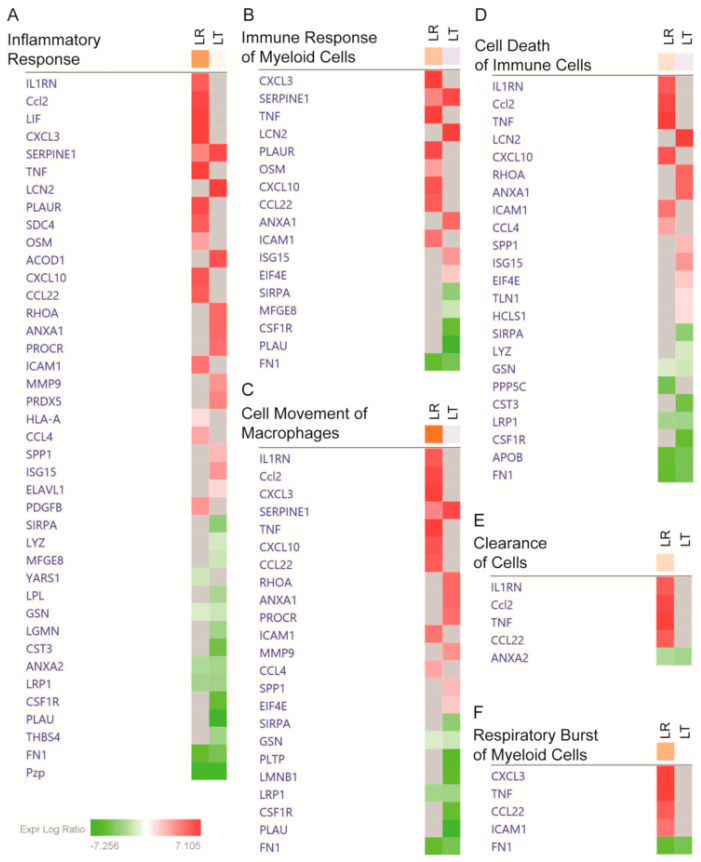
LPS-Responding RAW 264.7 cells’ secretomes include cytokines and signaling proteins strongly related to the inflammatory response and cell motility, while LPS-Tolerant RAW 264.7 cells’ secretomes include proteins strongly related to cell survival. Fold changes of the proteins associated by Ingenuity Pathway Analysis to Inflammatory Response (**A**); Immune Response of Myeloid Cells (**B**); Cell Movement by Macrophages (**C**); Cell Death of Immune Cells (**D**); Clearance of Cells (**E**), and Respiratory Burst of Myeloid Cells (**F**). Prepared using the Ingenuity Pathway Analysis program suite from QIAGEN (Germantown, MD, USA).

The canonical process associated with LPS treatment is the inflammatory response. While both LR and LT groups had highly significant effect changes in the inflammatory response (both *p*-values < 0.01), the LR group showed a strong increase (z-score of 1.908) and the LT group had a smaller increase (z-score of 0.204) (visualized in Figure 6A, Supplementary Table S2). When we focus on the myeloid cell responses, the differences in the LR and LT groups become even more striking. While the “Immune Response of Myeloid Cells” is significantly affected in either condition (*p*-values of <0.001), the LR condition had an increased response (z-score 1.134) but the LT condition had a decreased response (z-score −0.348) (visualized in Figure 6B, Supplementary Table S2). By examining a heatmap of the proteins measured from each condition, it was found that while the LR group had several signaling molecules, including CXCL3, CXCL10, and TNF, the LT group had decreased recovery of these signaling molecules along with decreased secretion (compared to the untreated control) of the urokinase-type plasminogen activator (PLAU) (Figure 6B), a secreted enzyme that activates plasmin, a protein that is critical for the complement system [30]. These results confirm that either type of LPS treatment induces the inflammatory response, but the response after sequential LPS treatment is significantly reduced.

Another biological function associated with all three sets and with the LPS response was cellular motility. Due to the variety of cells and mechanisms of movement, most analysis platforms include both general terms and specific pathways. In the LR group, “Cell Movement of Macrophages” was significantly increased (*p*-value < 0.001, z-score 2.829), and while the LT group had a highly significant increase (*p*-value < 0.001), the overall degree of migration was lower (z-score −0.290). In our comparison, both datasets were associated with migration and contained at least five significantly elevated or decreased proteins (Figure 6C). This suggests that both treatments lead to cellular migration, but the overall effect was much higher in the LPS-responsive group.

While the processes of inflammation and movement are critical for the immune response, cell survival has been the hypothetical goal of LPS tolerance. In support of this hypothesis, our results found significant inhibition of ”Cell Death of Immune Cells” in the LT group (*p*-value < 0.01, z-score −0.254) (Supplementary Table S2). In contrast, the LR group had a highly significant increase in the “Cell Death of Immune Cells” (*p*-value < 0.001, z-score 0.565) (Supplementary Table S2). The difference in the recovery of cell-survival-associated proteins from the LT and LR groups suggests a connection between cell survival and LPS tolerance (Figure 6D).

### 3.4. Relationship between Repeated LPS Stimulation and Cellular Exhaustion

**Figure 7:**
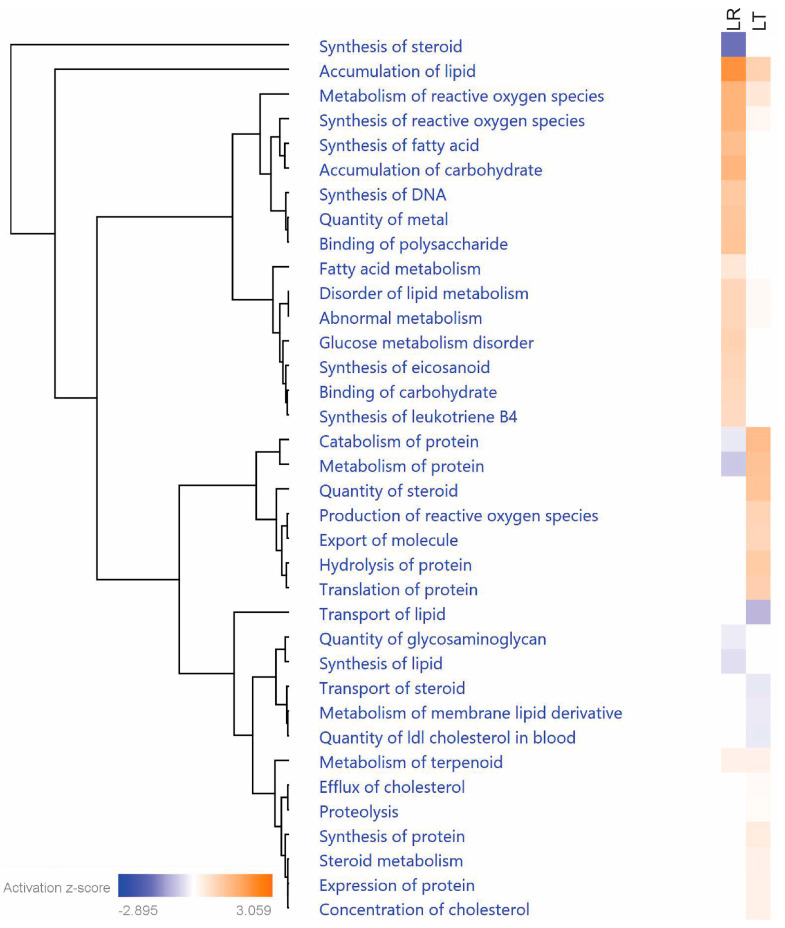
LPS-Tolerant RAW 264.7 cell secretomes include proteins related to initiation of protein metabolism. Comparison of the Ingenuity Pathway Analysis of proteins with significantly changed secretion in either LR or LT cells shows that while LR cells secreted proteins strongly relate to carbohydrate metabolism, LT cells secreted proteins that strongly relate to protein and reactive oxygen species metabolism. Prepared using the Ingenuity Pathway Analysis program suite from QIAGEN (Germantown, MD, USA).

We have hypothesized that cellular exhaustion is related to suppression of the LPS response in sequential LPS treatments. Two pathways that relate to exhaustion are metabolism and the production of reactive oxygen species. By filtering the IPA comparative analysis results only for processes related to metabolism or reactive oxygen species, we found distinct differences between the LR and LT groups (Figure 7). Overall, the LT group has a wide variety of affected processes, with both increased and decreased rates predicted.

**Figure 8:**
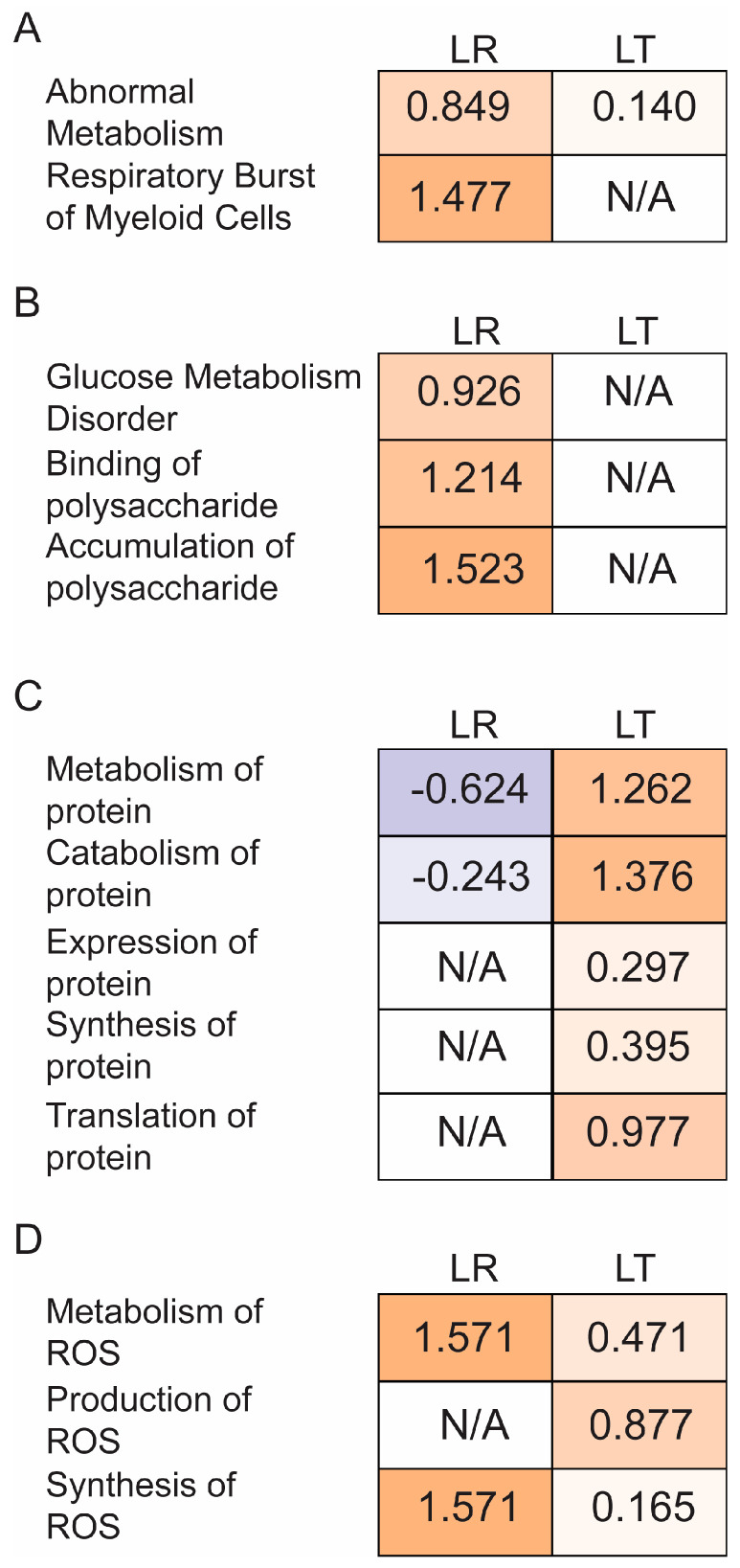
LPS-Tolerant RAW 264.7 cell secretomes include proteins related to global changes in cellular metabolism. z-scores of the metabolic pathway results generated by Ingenuity Pathway Analysis show abnormal metabolism and Respiratory Burst of Myeloid Cells in LR cells (**A**), increased carbohydrate metabolism by LR cells (**B**), increased protein metabolism along with decreased protein translation by LT cells (**C**), and increased maintenance of reactive oxygen species by both cells (**D**).

Metabolism can be further defined by the class of molecule targeted, such as protein, lipid, or carbohydrate. The two classes that exhibited the clearest differences between the LR and LT groups were the processes related to carbohydrate and protein metabolism. In carbohydrate metabolism, the overall effect is that the LPS response induced increased carbohydrate metabolism, including the binding, accumulation, and metabolism of polysaccharides (Figure 8B). In contrast to the carbohydrate results, an examination of the processes related to protein metabolism showed increased association between the LT group and protein metabolism. Overall, protein metabolism appears to lean towards the accumulation of new proteins, with the increased z-score of overall protein metabolism and protein synthesis coinciding with decreases in protein catabolism and proteolysis (Figure 8C). The last aspect of metabolism with distinct differences between the LR and LT groups is the pathways related to reactive oxygen species. Overall, the LT group results were linked to lower metabolism and synthesis of ROS compared to the LR group (Figure 8D, Supplementary Table S2). These results confirm the modifications in the cellular environment that occur during both the LPS response and LPS tolerance.

Based on the strong association of metabolism and reactive oxygen species with the previously shown effects of LPS tolerance on cellular respiration, we concluded that the induction and maintenance of LPS tolerance is dependent on the rates of cellular respiration, and further studies of the modifiers of cellular respiration and metabolic rates could lead to greater understanding of the regulation of LPS tolerance.

## 4. Discussion

LPS tolerance is a cellular condition defined by a lack of a typical immune response to LPS stimulation, originally characterized by decreased levels of secreted cytokines such as TNF-α, IL-6, and IL-10 (Figure 1). We have shown that LPS-tolerant RAW 264.7 cells secrete a wide variety of proteins, including several not typically found in the secretome, defined as proteins released from the cells as described by Koppenol-Raab et al. [19] (Figure 3 and Figure 4). Similarly, while LPS-responding cells have basal metabolic rates the same as or above unstimulated control cells, LPS-tolerant cells show a significant decrease in their glycolytic and aerobic respirations (Figure 2).

### 4.1. Most Evident Protein Level Changes in the Secretome

Using MS analysis combined with SILAC labeling to allow for direct comparisons of the Con (NT), LR, and LT secretomes, we identified global changes in the secretome following the induction of either the LPS response or LPS tolerance (Figure 3). It is important to note that the experimental setup with serum-free media necessary to facilitate mass spectrometry-based proteomics may affect the cell response. We have established that the cells respond to TLR ligands for up to 24 h, with the secretion patterns of known inflammatory cytokines being the same as the cells in the complete media [19], but there is a probability that some elements of the response to the second LPS stimulation, although many controls are as predicted for the LPS-tolerant state, may be changed by this variable. A comparison of the LR and LT secretomes further confirmed the vast differences in the quantity and types of secreted proteins that had significantly enhanced secretion (Figure 4). Using pathway analysis of the secreted proteins, we found that the LR cell secretome is highly associated with the innate immune response (Figure 5 and Figure 6). In contrast, the LT cell secretome is highly associated with cell survival and modulation of cellular metabolism (Figure 5, Figure 6 and Figure 7). These modulations focus on many aspects of both protein metabolism and reactive oxygen species metabolism (Figure 8).

### 4.2. Potential Protein Regulators of LPS Tolerance

The clear differences between LR and LT cells raise the question of which signaling molecules induce and maintain LPS tolerance following multiple stimulations with LPS. Possible inducers or regulators of LPS tolerance could be secreted proteins (previously shown by either the protein itself, a closely related protein, or a homolog), specifically enriched in the LPS-tolerant cells, that have been previously linked to two or three of the critical functions we identified above (cell survival, protein metabolism, and maintenance of ROS). An examination of the LT group proteins identified several proteins that fulfill many of these requirements (Table 1). Three proteins that were linked to all three of the critical functions were superoxide dismutase 2 (SOD2, just below the statistical significance threshold but important to mention), sequestosome 1 (SQSTM1), and osteopontin 1 (SPP1). In addition, three secreted proteins were specifically enriched and involved in cell survival along with redox. The last group of seven proteins have been shown to be secreted, were specifically enriched, and were involved in cell survival along with protein metabolism.

One protein with a direct association with the metabolism of protein and reactive oxygen species is mitochondrial superoxide dismutase (SOD2), whose deficiency has been linked to inflammatory disorders [31]. SOD2 has been shown to be increased in the process of the macrophage protection from reactive oxygen species-induced cell death [32]. Interestingly, its upregulation was described together with the downregulation of PARP1, an enzyme adding ADP-ribose to many proteins, a modification which we have recently shown to be regulated by LPS in macrophages [33]. Since we have found many proteins involved in the inhibition of apoptosis and necrosis in LPS-tolerant cells, there may be crucial mechanisms affected by proteins within this group that can be targeted for tolerance induction.

The second secreted protein that affects all three processes is sequestosome 1 (SQSTM1, or p62), a receptor for selective autophagy that is responsible for sequestering cytoplasmic components into an autophagosome [34] and which, by its role in regulation of autophagy, affects macrophage survival. Because of these roles in autophagy, its upregulation following LPS tolerance would be another indication of the switch to survival mode. Additionally, SQSTSM1/p62 has been proposed to act as an inflammatory signaling platform after activation by transforming growth factor beta-activated kinase 1 (TAK1) (one of the kinases essential in TLR4 signaling [35]), effectively disabling it as an autophagy receptor and inhibiting its own degradation [36].

The final secreted protein that affects all three processes is osteopontin 1 (SPP1), a secreted bone matrix glycoprotein protein that is essential for bone homeostasis and control of cell migration [37,38]. SPP1 has also been shown to be expressed by macrophages during tissue repair after myocardial infarction [39], indicating its function in the tissue homeostasis function of macrophages as opposed to the inflammatory function. The secreted proteins may also provide an autocrine signal to balance cytokine production, a main feature of LPS tolerance. For example, in LT, the IL-1 receptor antagonist (Table 1) might directly decrease cytokine production [40], while Lipocalin-2 counteracts LT through the induction of cytokine production [15]. Hence, the understanding of these proteins and complex feedback loops is fundamental to control LPS signaling and macrophage function.

The specificity of the secreted proteins associated with LPS tolerance does raise the question of the role of the regulation of protein signaling in leading to stimulation type-specific protein secretion in the initiation and maintenance of LPS tolerance. The role of post-translational modifications, especially phosphorylation, was pointed out as a regulatory mechanism in LPS tolerance nearly thirty years ago [41] and linked to crosstalk with other signaling pathways, for example, Fc gamma receptors (FcGRs) [42]. On the other hand, pathways intuitively associated with the regulation of the immune response may not be required for the induction of endotoxin tolerance, as shown for type 1 interferon signaling [43]. In addition to TLR4 signaling, NLRP3 inflammasome has been shown to play an important role in the response to LPS and has recently been shown to be regulated by specific lipid mediators [44]. These results may open another avenue for exploration of the mechanisms of LPS tolerance and for explanation of changes in the secretome. Cellular metabolism has recently emerged as a regulator of macrophage phenotype in general [45]. Unbiased secreted protein profiling and system-level characterization of changes in innate immune signaling and cellular metabolism, pointing to regulation at the post-transcriptional level, emphasize the importance of global studies that reach beyond gene expression analysis. Our study reveals the value of proteomics approaches that can explain rapid functional changes necessary for effective immune function.

In the clinic, macrophage LPS tolerance could be either beneficial or harmful to the host, depending on other factors. While well-controlled LPS tolerance reduces overwhelming cytokine production (cytokine storm) and attenuates sepsis severity [46], unhinged LPS tolerance immune exhaustion might be harmful [47]. Novel ways to inhibit cytokine secretion and controlled induction of LPS tolerance should therefore be considered as a future treatment of septic shock.

## Figures and Tables

**Table 1:** Potential regulators of LPS tolerance. Proteins with enhanced secretion by LPS-Tolerant RAW 264.7 cells vs. control and LPS-Responding cells that have associations with cell survival along with associations with either protein metabolism, reactive oxygen species metabolism, or both. “Secreted” results include “Yes” for proteins shown previously to be secreted in mice, “Yes (related)” for proteins shown previously to be secreted in humans, or “Yes (exosome)” for proteins shown previously to be secreted in exosomes in humans.

Uniprot	Protein Name	Entrez Gene Name	LT Enrichment/LR Enrichment	Secreted	Cell Survival	Protein Metabolism	Redox
**P09671**	SOD2	superoxide dismutase 2	3.7913	Yes (exosome)	Yes	Yes	Yes
**Q64337**	SQSTM1	sequestosome 1	4.6730	Yes (exosome)	Yes	Yes	Yes
**P10923**	SPP1	secreted phosphoprotein 1	2.3679	Yes	Yes	Yes	Yes
							
**Q62351**	TFRC	transferrin receptor	4.7225	Yes	Yes	No	Yes
**P60710**	ACTB	actin beta	3.6136	Yes (related)	Yes	No	Yes
**P99029**	PRDX5	peroxiredoxin 5	5.3956	Yes (related)	Yes	No	Yes
							
**Q3TCH7**	CUL4A	cullin 4A	1.9345	Yes (exosome)	Yes	Yes	No
**Q9CXW4**	RPL11	ribosomal protein L7	1.1280	Yes (exosome)	Yes	Yes	No
**P46471**	PSMC2	proteasome 26S subunit, ATPase 2	1.6182	Yes (related)	Yes	Yes	No
**P11438**	LAMP1	lysosomal associated membrane protein 1	2.8676	Yes (exosome)	Yes	Yes	No
**P22777**	SERPINE1	serpin family E member 1	2.2662	Yes	Yes	Yes	No
**P25085**	IL1RN	interleukin 1 receptor antagonist	1.0980	Yes	Yes	Yes	No
**P41245**	MMP9	matrix metallopeptidase 9	3.7096	Yes	Yes	Yes	No

## Data Availability

The mass spectrometry-based proteomics data have been deposited to the ProteomeXchange Consortium via the PRIDE partner repository with the dataset identifier PXD021925.

## Supplementary Materials

The following are available online at https://www.mdpi.com/2218-273X/11/2/164/s1, Supplementary Table S1. Sheet 1: All of the proteins identified in all the analyses (protein identifiers: Column C). Sheet 2: Protein result filtering to identify proteins with two or more peptides and 12 or more valid values. Sheets 3–5: Imputation of missing values and conversion of data into Log2 Fold changes in protein intensity recorded. Sheet 6: Summary of protein quantification and *p*-values for all the proteins quantified. Supplementary Table S2. All of the biological processes and cellular functions examined in the LPS-Responding and LPS-Tolerant cells, with *p*-values and significance marked with asterisks (increasing significance is indicated with more asterisks).
